# Annotations

**Published:** 1905-05-27

**Authors:** 


### ANNOTATIONS.

### Hospital Courtesies.

In hospital practice, just as in private practice, it not infrequently happens that the patient in different stages of his illness comes under the care and observation of different practitioners. More especially, of course, is this the case in chronic disorders, sufferers from which notoriously prefer, in many instances at least, to seek admission to another hospital rather than to return to the one in which they were formerly treated. This habit certainly means the accumulation of many broken and incomplete clinical records which must, in the nature of things, possess but little scientific and practical value. Such defects would be largely minimised were the practice adopted of establishing habitual communication between the hospitals successively concerned with such cases. This plan, in principle and theory at least, is recognised as an appropriate procedure in private practice, and it might suitably be extended as a routine method amongst those responsible for hospital patients. Such a custom would be for the benefit of all concerned. The physician or surgeon, as the case might be, would gain a much enlarged experience of the natural histories of his cases and of disease processes in general, and this would apply equally to those seeing a case in its earlier phases as well as to those observing its later developments. Valuable as this would be even to senior members of the staff it would be particularly serviceable to those of junior experience, on whom the mechanical labour of communications would chiefly fall. The general knowledge of the movements of disease, more particularly in its chronic forms, would be made more confident and sooner or later this would doubtless lead to improvements in treatment and to the greater relief of the individual sufferer. These and other benefits would certainly accrue were a more frequent habit of mutual clinical communication established between hospitals and similar institutions.

### The Prevention of Glanders.

What is the use of a trustworthy test for detecting the presence of a destructive disease unless it be applied? We make this inquiry because at an inquest on Saturday last, the deceased being a horsekeeeper who had died from glanders, it was pointed out by the representative of the London County Council that the mallein test by which it can be ascertained whether a horse has glanders or not is not compulsory. It should, of course, be voluntarily resorted to whenever there is the slightest cause for suspicion that the disease exists. It is true that glanders in man is rare, but that is no reason why precautions should not be aimed at. And this is not only on humanitarian grounds, but also necessary in the economic interests of the community. In the report for 1904 on the diseases of animals there was a most satisfactory decrease in the prevalence of nearly every infectious illness to which domestic animals are liable. The notable exceptions were anthrax and glanders, both of which showed a decided increase during 1904. The figures for glanders show a total for that year of 1,563 recorded outbreaks, the number of horses attacked being 2,658. For the majority of these outbreaks, the metropolitan area was responsible, no fewer than 1,080 having occurred in the County of London, and 192 in the neighbouring counties of Middlesex, Surrey, and Essex. We give the figures because they show how much loss is entailed upon the community through failure to exercise rational and adequate safeguards against the spread of glanders. In the instance under consideration it had been prevalent for some months in the stable, and since the horsekeeper's death the application of the mallein test to the entire stock has resulted in the discovery that seven horses in addition to those already killed were infected. They were destroyed also; but it is impossible to know to what extent they communicated the malady while they were alive. If, when the disease first appeared, the test had been applied to the entire stud, the risks of infection would have been reduced to a minimum, and, for the sake of beast and man, it is of vital importance that owners of horses should cease to possess the option of neglecting an investigation which ought to be obligatory, with a heavy penalty in the event of neglect.

### Control of the Feeble-Minded.

Although the feeble-minded child has now all necessary care and protection, the powers possessed by Boards of Guardians for dealing with feeble-minded persons, not certifiable as idiots or imbeciles, are substantially the same as those they possess with normal individuals. It is obvious that such limited powers do not meet the needs of the case. The Guardians have disciplinary powers over feeble-minded inmates of any institution under their own control, but they have not the power to prevent such inmates taking their discharge when they choose. Now, the feeble-minded person is like a child, capable in many cases of leading a useful and happy life while guided by a superior will and intelligence, but very ill-fitted to guide his own path through the mazes of the outside world. The feeble-minded youth finds his way to the police-court, though he is but the catspaw of cleverer criminals. The feeble-minded girl has only too often to seek refuge in the maternity ward of the workhouse. Both contribute to the reproduction of the unfit. Guardians have power to send a feeble-minded mother and her child to any institution where they can both be received, and, if the consent of the Local Government Board be obtained, to pay for them out of the rates. But there is no power to prevent them leaving when they will, if only they consent to take their children with them, and in many cases restlessness or mere unconscious and irresponsible passion induces them to return to that world with which they are so ill-fitted to battle. These return again and again to the workhouse to give birth to illegitimate children. Such cases are known to all who do Poor-law work, and much stronger powers are needed for dealing with them. It is therefore to be hoped that the special efforts which are now being made by the National Association for Promoting the Welfare of the Feeble-minded may succeed in arousing the public from its habitual apathy towards one of the most pressing social problems of to-day.

